# Leveraging deep learning for plant disease and pest detection: a comprehensive review and future directions

**DOI:** 10.3389/fpls.2025.1538163

**Published:** 2025-02-21

**Authors:** Muhammad Shoaib, Abolghasem Sadeghi-Niaraki, Farman Ali, Irfan Hussain, Shah Khalid

**Affiliations:** ^1^ Department of Computer Science, CECOS University of IT and Emerging Science, Peshawar, Pakistan; ^2^ Department of Computer Science and Engineering and Convergence Engineering for Intelligent Drone, XR Research Center, Sejong University, Seoul, Republic of Korea; ^3^ Department of Applied AI, School of Convergence, College of Computing and Informatics, Sungkyunkwan University, Seoul, Republic of Korea; ^4^ Centre for Autonomous Robotic Systems, Khalifa University, Abu Dhabi, United Arab Emirates; ^5^ School of Electrical Engineering and Computer Science, National University of Sciences and Technology, Islamabad, Pakistan

**Keywords:** plant disease, pest detection, deep learning, CNNs, agri-tech, computer vision

## Abstract

Plant diseases and pests pose significant threats to crop yield and quality, prompting the exploration of digital image processing techniques for their detection. Recent advancements in deep learning models have shown remarkable progress in this domain, outperforming traditional methods across various fronts including classification, detection, and segmentation networks. This review delves into recent research endeavors focused on leveraging deep learning for detecting plant and pest diseases, reflecting a burgeoning interest among researchers in artificial intelligence-driven approaches for agricultural analysis. The study begins by elucidating the limitations of conventional detection methods, setting the stage for exploring the challenges and opportunities inherent in deploying deep learning in real-world applications for plant disease and pest infestation detection. Moreover, the review offers insights into potential solutions while critically analyzing the obstacles encountered. Furthermore, it conducts a meticulous examination and prognostication of the trajectory of deep learning models in plant disease and pest infestation detection. Through this comprehensive analysis, the review seeks to provide a nuanced understanding of the evolving landscape and prospects in this vital area of agricultural research. The review highlights that state-of-the-art deep learning models have achieved impressive accuracies, with classification tasks often exceeding 95% and detection and segmentation networks demonstrating precision rates above 90% in identifying plant diseases and pest infestations. These findings underscore the transformative potential of deep learning in revolutionizing agricultural diagnostics.

## 1 Introduction

Researchers are using image data to detect diseases and pest infestation in plants using machine learning models and computer vision techniques. Detection of diseases and pest infestation in plants is a technique of photographing plants with industrial vision equipment to check for diseases or pests (Shoaib et al., 2023). In this method, the shooting angle and light source are selected according to how the diseases and pests behave, so that a uniformly illuminated photo can be created. Computer-vision-based methods combined with traditional image processing techniques and manual feature design and classifiers are commonly used to detect plant diseases and pest infestation (Xiong et al., 2024).

On the other hand, well-designed imaging schemes can significantly reduce the complexity of developing traditional algorithms while also increasing implementation costs. Simultaneously, expecting traditional algorithms to eliminate scene changes from recognition results in the natural environment is often unrealistic (Hurtado et al., 2023). A complex natural environment presents numerous challenges. Lesion areas differ modestly from their contexts, contrast is low, the size of the lesion area differs significantly, and noise levels in the image of the lesion are variable. In natural light, it is difficult to detect plant diseases and pest infestations because of numerous distractions. In this situation, traditional methods are often ineffective and ineffective.

During the last few years, the effective implementation of deep learning models based on convolutional neural networks (CNN) has improved several computer vision (CV) applications, including traffic detection (Karvelis et al., 2020), recognition of medical images (Mehmood et al., 2018), text recognition in scenarios (Moustafa et al., 2019), facial expression detection (Khare et al., 2024), and identification of faces (Shah et al., 2022). Wechat disease and pest infestation detection apps based on deep learning and photo recognition APP applications have been developed by several national and international companies. As a result, methods based on deep learning for detecting plant diseases and pest infestation have significant value in both academic and commercial fields.

Recent studies have made significant advancements in plant disease detection and agricultural prediction using cutting-edge technologies. One such study (Vishnoi et al., 2023), demonstrated the effectiveness of CNNs in identifying diseases in apple plants based on leaf images, showcasing how deep learning can automate and enhance disease diagnosis. Another important work (Joshua et al., 2022), explored various machine learning models for predicting crop yields across different types of crops, emphasizing the importance of data-driven approaches in improving agricultural productivity. Additionally, the study (Khan et al., 2022) introduced a novel IoT-based system that uses environmental data to recommend precise fertilizer application, highlighting how IoT can optimize resource usage and improve crop health. The study (Parashar et al., 2024) provided a thorough review of various yield prediction models, analyzing their advancements and the challenges that still need to be addressed for more accurate and reliable predictions. Together, these studies contribute to the growing body of research that blends machine learning, IoT, and data analytics to revolutionize agriculture and enhance sustainability.

This study makes several significant contributions to the field of plant pathology and agricultural science:

- It provides a comprehensive review of recent advancements in the application of deep learning techniques for the detection of plant diseases and pests. By synthesizing and analyzing the latest research findings, the study offers valuable insights into the strengths and limitations of deep learning models in addressing this critical agricultural challenge.
- The study identifies and elucidates the challenges associated with the adoption of deep learning methods in real-world agricultural settings. By highlighting issues such as dataset scarcity, model complexity, and detection speed, the study offers researchers and practitioners a roadmap for overcoming these obstacles and advancing the state-of-the-art in plant disease and pest detection.
- The study proposes potential solutions to address the identified challenges, including data augmentation techniques, transfer learning strategies, and the development of lightweight network architectures. By outlining these solutions, the study not only informs future research directions but also empowers practitioners to implement more effective and efficient detection systems in agricultural settings.

The article begins with an Introduction, highlighting the significance of plant disease and pest detection in agriculture and the role of advanced technologies like deep learning in addressing these challenges. The second section, Pest and Disease Detection Problems in Plants, explores the common issues associated with plant diseases and pests, along with the limitations of traditional detection methods. The third section, Deep Learning for Image Recognition, provides an overview of deep learning techniques such as CNNs and Transformers, emphasizing their impact on image recognition in agriculture. The fourth section, Deep Learning for Plant Disease Detection, focuses on recent advancements in classification, detection, and segmentation models tailored for plant disease identification. The fifth section, Integration of IoT and Edge Computing for Enhanced Plant Disease Detection, discusses the potential of IoT and edge computing technologies to complement deep learning models for real-time agricultural diagnostics. In the sixth section, Analyzing the Dataset and Comparing its Performance, the importance of robust datasets and a comparative analysis of model performance are presented. The seventh section, Evaluation Indices, explains the metrics used to assess the effectiveness of deep learning models. The eighth section, Challenges, addresses the obstacles in deploying these technologies in real-world scenarios. Finally, the ninth section, Future Directions, outlines potential research opportunities and innovations to advance plant disease and pest detection using deep learning.

## 2 Pests and disease detection problems in plants

### 2.1 An overview of plant disease and pest definitions

Natural disasters such as plant diseases and pests threaten plants at all stages of their life cycle, from seed production to seeding and seedling development. The concept of plant diseases and pests is often used instead of mathematics when it comes to industrial vision tasks. As a result, automating the task becomes more difficult. In order to simplify the task, it can be divided into simpler parts, such as detecting individual pests or diseases. Labeled training data can be used to train the system (Maity et al., 2023).

### 2.2 Detection of plant diseases and pests

**Figure 1:**
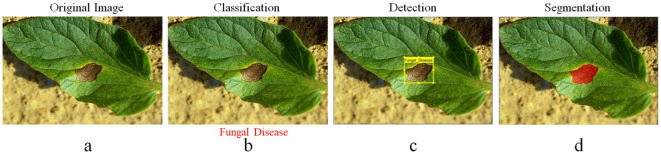
**(a)** Input raw image, **(b)** leaf classification, **(c)** lesion detection, and **(d)** lesion segmentation.

Pest infestations require broader criteria for detection, identification, and segmentation (Kiobia et al., 2023). Its criteria are divided into three groups: what, where, and how, with “who” referring to the first-level classification role in computer vision. The group to which it belongs, as shown in **Figure 1**, is indicated. This step is known as classification, and it consists solely of providing information about the image category. In computer vision, the “where” on the second level is a representation of location, and the “where” on the third level is a representation of robust detection. This step determines whether an image contains any sign of diseases or pests in plants, as well as where they are located. In **Figure 1**, a rectangular box denotes the grey mould plate area. The “Why” task in computer vision is the third-level segmentation task. As shown in **Figure 1**, grey mould lesions are separated from the background pixel by pixel, allowing for the extraction of additional data such as the duration, region, and position of grey mould lesions, which can help assess diseases and pest severity infestation in plants.

Object detection involves analyzing the local features of an object to determine its spatial location within an image. This process primarily relies on machine learning techniques, which consider attributes such as shape, size, and color (Ma et al., 2023). Its applications span diverse domains, including autonomous vehicles, medical image analysis, and security systems. Classification, an integral facet of object detection, entails globally describing an image through feature representations, followed by classification operations to ascertain the presence of specific objects. This method ensures a comprehensive understanding of the image content before making object-specific determinations. The process of detecting plant diseases and pest infestations encompasses three distinct stages, each serving a unique function but interconnected and transformative in nature. The initial stage focuses on identifying the presence of relevant objects within the image (“what” task), followed by determining their spatial location (“where” task). Lastly, the process concludes with understanding the nature of these objects (“how” task), which further refines the detection process. It is also noteworthy that specific approaches may be employed to achieve the objectives of the second and third stages by leveraging the outcomes of the initial stage. For the sake of clarity and consistency, the term “identifying plant diseases and pest infestations” is employed throughout this discourse, with variations in network structures and functionalities being the sole distinguishing factors in terminology.

From four perspectives, including gasoline, we compare traditional methods of detecting plant and pest diseases based on existing references (Joseph et al., 2023), to better illustrate the characteristics and application scenarios of detection methods for plant diseases and pest infestations. The results of the comparison are presented in **Table 1**.

**Table 1:** A comparison is made between traditional image processing methods and deep learning techniques.

Aspect	Traditional DIP Methods	Cutting-Edge Deep Learning
Foundation	Manual feature crafting, rule-based	Autonomous learning from data
Features Extraction	Handcrafted features, SIFT, HOG, LBP	CNNs, attention mechanisms, transfer learning
Classification methods	SVM, Backpropagation, Bayesian methods	End-to-end learning, hierarchical deep networks
Advance Techniques	Scale-Invariant Feature Transform (SIFT), Histogram of Oriented Gradients (HOG), Local Binary Patterns (LBP)	Attention mechanisms, transfer learning, pre-trained models
Model Adaptability	Relies on manually crafted features, may struggle with complex patterns	Learns intricate features autonomously, superior adaptability to dynamic environments
Enhancements for Classification	Support Vector Machines (SVM), Backpropagation (BP), Bayesian methods	End-to-end learning, exploiting hierarchical nature, ensemble methods
Preprocessing Tools	Limited preprocessing tools, often manual	Batch normalization, dropout layers, activation functions for enhanced robustness
Performance Optimization	Utilizes advanced tools for nuanced decision-making	Leverages data augmentation, transfer learning, and ensemble methods for optimal performance
Coexistence with Deep Learning	Coexists but relies on manual feature engineering	Represents a paradigm shift, offering superior adaptability and automation
Decision Factors	Suitable for scenarios with limited data, computational constraints	Ideal for large datasets, computational resources, dynamic imaging environments
Impact on Imaging Landscape	Traditional methods are still relevant but facing challenges	Marks a paradigm shift, transforming the landscape of image processing
Considerations for Choice	Data volume, computational resources, simplicity vs. complexity	Data volume, computational resources, adaptability to dynamic imaging environments

## 3 Deep learning image recognition

A deep learning model-based image recognition technique does not require the extraction of specific features, rather it iteratively discovers the most suitable ones. Image features can be extracted globally and contextually, with high accuracy and robustness.

### 3.1 Deep learning theory

“Deep Learning” was popularized in a 2006 Science article by Kabir et al (Kabir et al., 2023). Multiplying hidden layers is the basic principle of deep learning. Perceptrons serve as hidden layers; perceptrons extract low-level characteristics, and low-level characteristics are combined to achieve high-level abstract characteristics, thereby minimizing the local minimum problem. Using deep learning, researchers can overcome the shortcomings of traditional algorithms that rely on artificial features. As a result, it has been applied to many areas such as computer vision, face recognition, speech recognition, natural language processing, recommendation systems, and speech recognition (Chan et al., 2023). This stumbling block can also be removed using the deep learning method. It can use unsupervised learning to extract information on low-level, intermediate-level, and high-level semantic characteristics directly from the original image. Traditional plant disease and pest infestation detection algorithms are based on manually designed features extracted from original images. Human intervention is not required for deep learning algorithms. With robust autonomous learning and characteristic expression abilities, this model comprises several layers that can automatically classify and recognize images. Therefore, deep learning can be used to identify diseases and pests in photographs. This technique has been used to create several well-known deep neural network models. A few of them are Deep Belief Networks (DBN) (Ahmed et al., 2023), Boltzmann’s Deep Machines (DBMs) (Liu et al., 2017), Battery Noise Delete Autoencoders (SDAEs) (Sun et al., 2023), and Deep CNNs (Li et al., 2022). Image recognition techniques based on deep neural networks have several advantages over manual methods for extracting features from a space of prominent features. With more learning samples and computational power, deep neural networks become better at analyzing data. A wide margin separates deep neural networks from traditional models in academia and industry. Among deep learning environments, deep convolution neural networks have recently gained popularity.

### 3.2 Artificial neural network based on convolution

**Figure 2:**
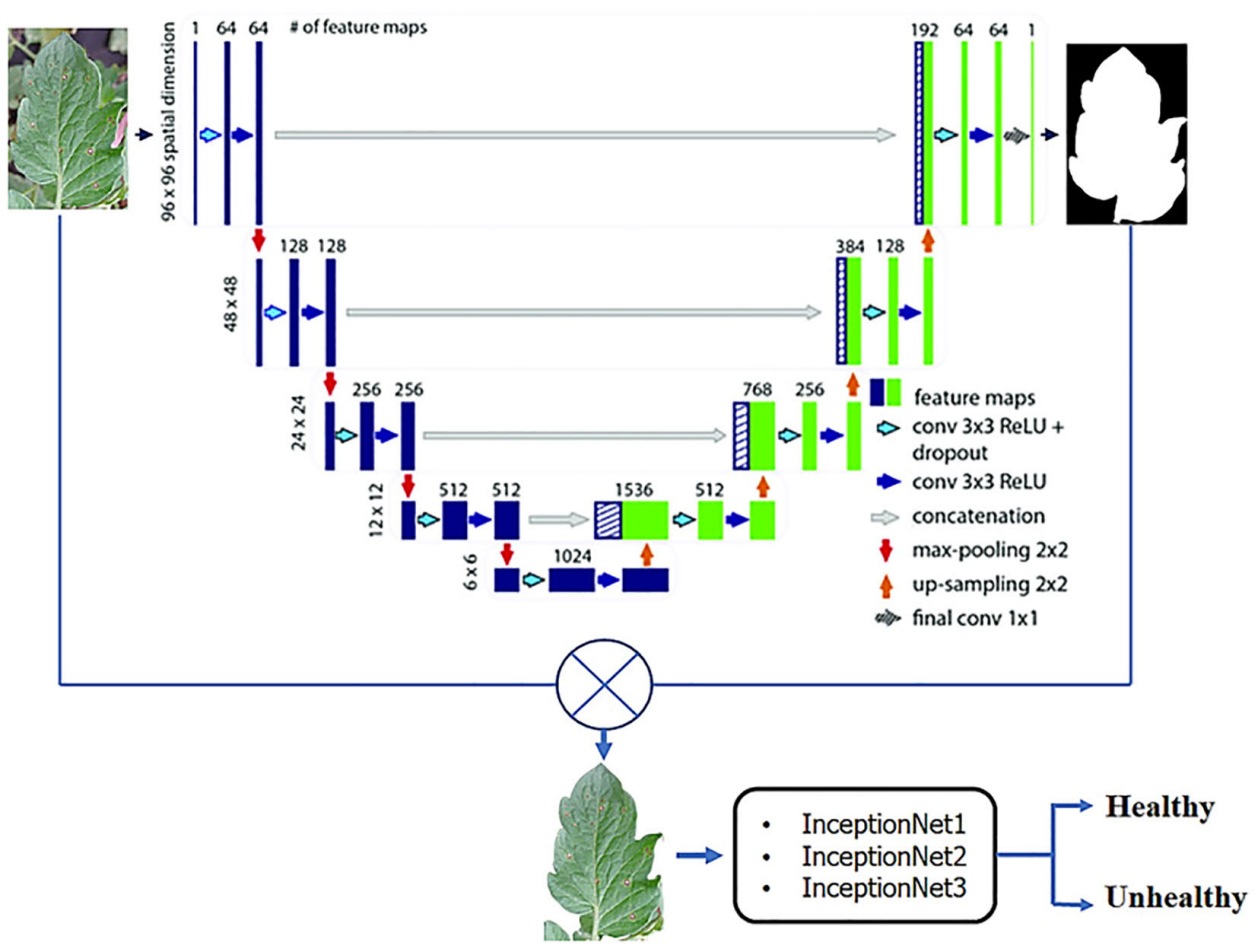
A CNN framework for classifying plants into healthy and unhealthy (Shoaib et al., 2022).

Learning-based features are extracted by convolutional neural networks and image classification is performed by these networks (see **Figure 2**). One of the most well-known deep learning models is CNN. The network’s essential structural features provide CNN with a significant advantage in image recognition because they provide a great deal of model capacity and complex information.

While CNN has been successful in computer vision tasks, deep learning has seen an increase in popularity. First, the convolution layer defines a convolution nucleus. In addition to its local receptive field, the convolution neural network has several other advantages. Convolution cores slide across feature maps to extract some of the information from them. To group local receptive field values, average, maximum, and random values are calculated (Alzubaidi et al., 2021).

### 3.3 Toolkits for deep learning based on open-source software

TensorFlow (Zainab et al., 2019), Torch/PyTorch (C. Science et al., 2021), Cafe (Shoaib et al., 2022), and Teano (Alzubaidi et al., 2021) are some of the most widely used open-source third-party deep learning tools. The features of each open-source tool are listed in **Table 2**. The four most popular open-source learning libraries are cross-platform, meaning they can run on Ubuntu, Windows, iOS, Android, and other platforms. On systems with high-end GPUs, PyTorch and TensorFlow with Keras can efficiently form large CNN networks, and are extremely stable libraries that support numerous third-party libraries.

**Table 2:** Software for deep learning based on open-source sources.

Techniques	Editors	Hardware Compatibility	Languages	Usability
TensorFlow	Google	CPU, GPU, Mobile	C++, Python, Java	Develop flexible applications, port to different platforms, increase performance, and support distributed applications.
Deeplearning4j	Skymind	CPU, GPU	Java	Distributed deep learning, supports integration with Hadoop and Spark.
MATLAB	MathWorks	CPU, GPU	MATLAB	Comprehensive support for deep learning and computer vision, extensive toolbox, and functions for image processing.
R Language	R Foundation	CPU, GPU	R	A growing ecosystem for deep learning, various packages for computer vision tasks, statistical analysis, and visualization.
Theano	MILA	CPU, GPU	C++, Python, C#	High level of performance and flexibility.
Torch	Face	CPU, GPU, FPGA	C#, Python, Lua	Simple debugging and development support dynamic neural networks. Easily expandable, modularized, and cost-effective learning.

### 3.4 Leveraging GANs and VAEs to address data scarcity

One of the critical challenges in applying deep learning to plant disease and pest detection is the scarcity of diverse and high-quality datasets. This issue is particularly significant in agriculture, where variations in environmental conditions, crop species, and disease presentations make it difficult to build comprehensive datasets. Generative Adversarial Networks (GANs) and Variational Autoencoders (VAEs) have emerged as powerful tools to overcome this limitation by generating synthetic data that mimics real-world variations, thereby augmenting existing datasets and improving model performance. GANs consist of two neural networks—a generator and a discriminator—that work in tandem. The generator creates synthetic images, while the discriminator evaluates their authenticity against real images, gradually pushing the generator to produce increasingly realistic outputs. In the context of plant disease and pest detection, GANs can simulate diverse lesion patterns, pest appearances, and environmental conditions such as lighting and background variations. These synthetic images enrich the dataset and improve the robustness of deep learning models, ensuring they perform well across different scenarios.

VAEs on the other hand, learn latent representations of the data, enabling the generation of new, plausible samples by interpolating between existing data points. VAEs are particularly useful for producing variations of plant images that maintain the underlying features of diseases or pests while introducing subtle differences. This ability to generate realistic variations can address the imbalance in datasets by increasing the representation of under-represented classes, such as rare diseases or pests. The integration of GANs and VAEs into the data preparation pipeline offers several advantages. First, these models significantly reduce the dependency on large, annotated datasets, which are time-consuming and expensive to collect in agricultural settings. Second, they enable researchers to simulate specific conditions, such as early-stage disease symptoms or variations across different crop types, to improve model generalization. Finally, the use of synthetic data generated by GANs and VAEs can enhance the scalability of plant disease detection systems, making them applicable to a broader range of crops and environmental conditions. Recent studies in related fields, such as biomedical imaging and natural image synthesis, have demonstrated the efficacy of GANs and VAEs in augmenting datasets and improving model performance. Their application in agriculture is still emerging but holds immense potential for advancing plant disease and pest detection systems. By leveraging these generative models, researchers can develop more robust, efficient, and scalable solutions to address the pressing challenges in agricultural technology.

## 4 Deep learning for plant disease detection

**Figure 3:**
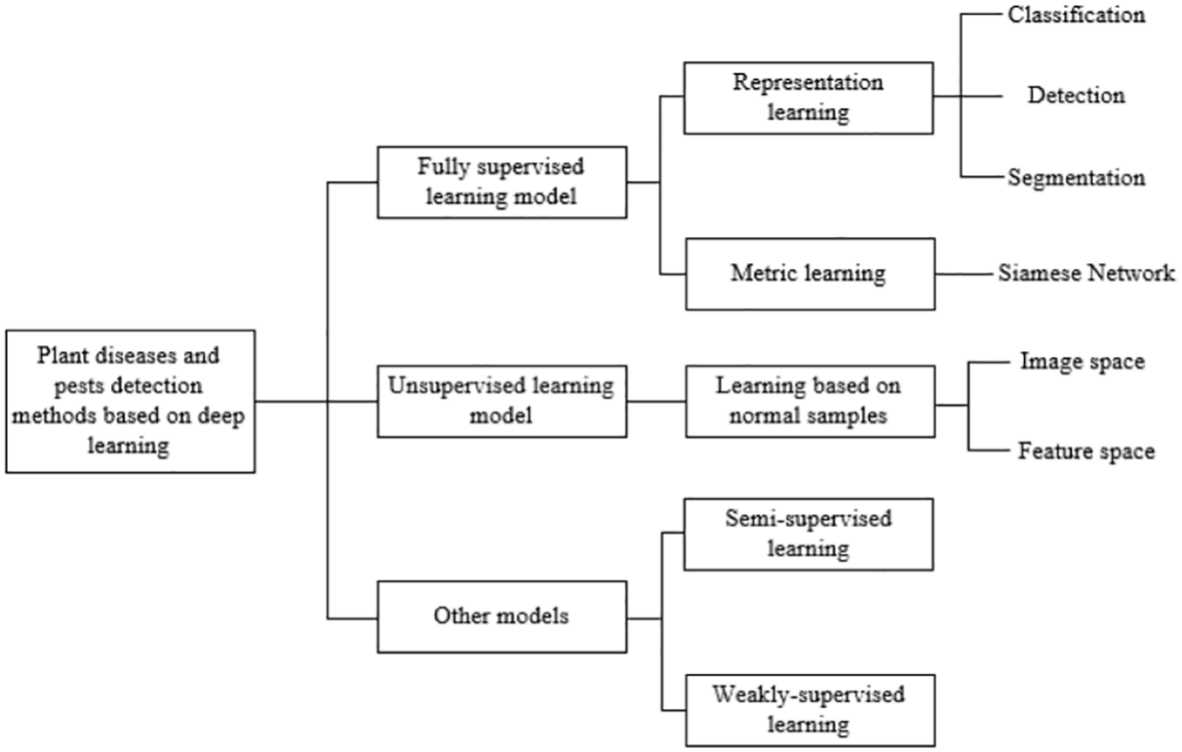
A general framework for plant and pest detection methods based on artificial intelligence.

This section addresses the early detection of plant diseases and pest infestations, emphasizing the integration of deep learning methodologies into traditional agricultural networks (Kaya and Gürsoy, 2023). The objective aligns seamlessly with computer vision tasks, facilitating the adaptation of deep learning techniques for the identification of plant and pest ailments. **Figure 3** delineates classification, detection, and segmentation networks, elucidating their respective network structures. Subsequently, a comprehensive summary of the distinctive attributes inherent to each methodological approach is provided, as depicted in **Figure 3**.

Explainable Artificial Intelligence (XAI) enhances plant disease and pest detection by addressing the “black-box” nature of deep learning models, making their predictions interpretable and transparent. Tools such as SHAP, LIME, and Grad-CAM allow visualization of key features, like leaf texture or discoloration, contributing to model predictions, enabling farmers to verify and trust AI-driven decisions. XAI facilitates informed decision-making by highlighting critical areas in images for disease treatment and pest management while identifying biases in training data to improve model generalization across diverse agricultural settings. Additionally, transparent AI systems align with regulatory compliance, foster adoption, and can integrate with IoT devices for real-time monitoring, offering actionable insights for precision-driven agriculture.

### 4.1 Network of classifications

In the real world, detecting plant diseases and pest infestations can be challenging due to differences in shapes, sizes, textures, colors, backgrounds, layouts, and lighting (Shoaib et al., 2023). Plant and pest diseases are classified using CNN-based classification networks because of their high capacity to extract characteristics. Some studies (Rai and Pahuja, 2024) have developed a network architecture based on real-world problems. A new test image is analyzed, and a label is assigned to the image categories in that class when added to the classification model. The classification network method is divided into two subcategories based on the tasks it performs: 1) CNN as a characteristics descriptor and 2) CNN as a decision-making system for detecting and locating lesions in plants.

### 4.2 Extraction of features from the network

Early deep-learning-based disease and pest classification methods were able to take advantage of CNN’s powerful characteristic extraction capability. A combination of the techniques was combined with an approach based on machine learning (Diana Andrushia et al., 2023). A CNN-based meta-architecture with characteristic extractors was proposed by Simbiring et al (Sembiring et al., 2023). by crossing meta-architectures. An SVM linear multiclass model was trained using features and labels extracted from the nine types of rice diseases by Mendoza et al (Mendoza-Bernal et al., 2024). It had a validation accuracy of 97.5%.

### 4.3 Classifying directly using a network

**Figure 4:**
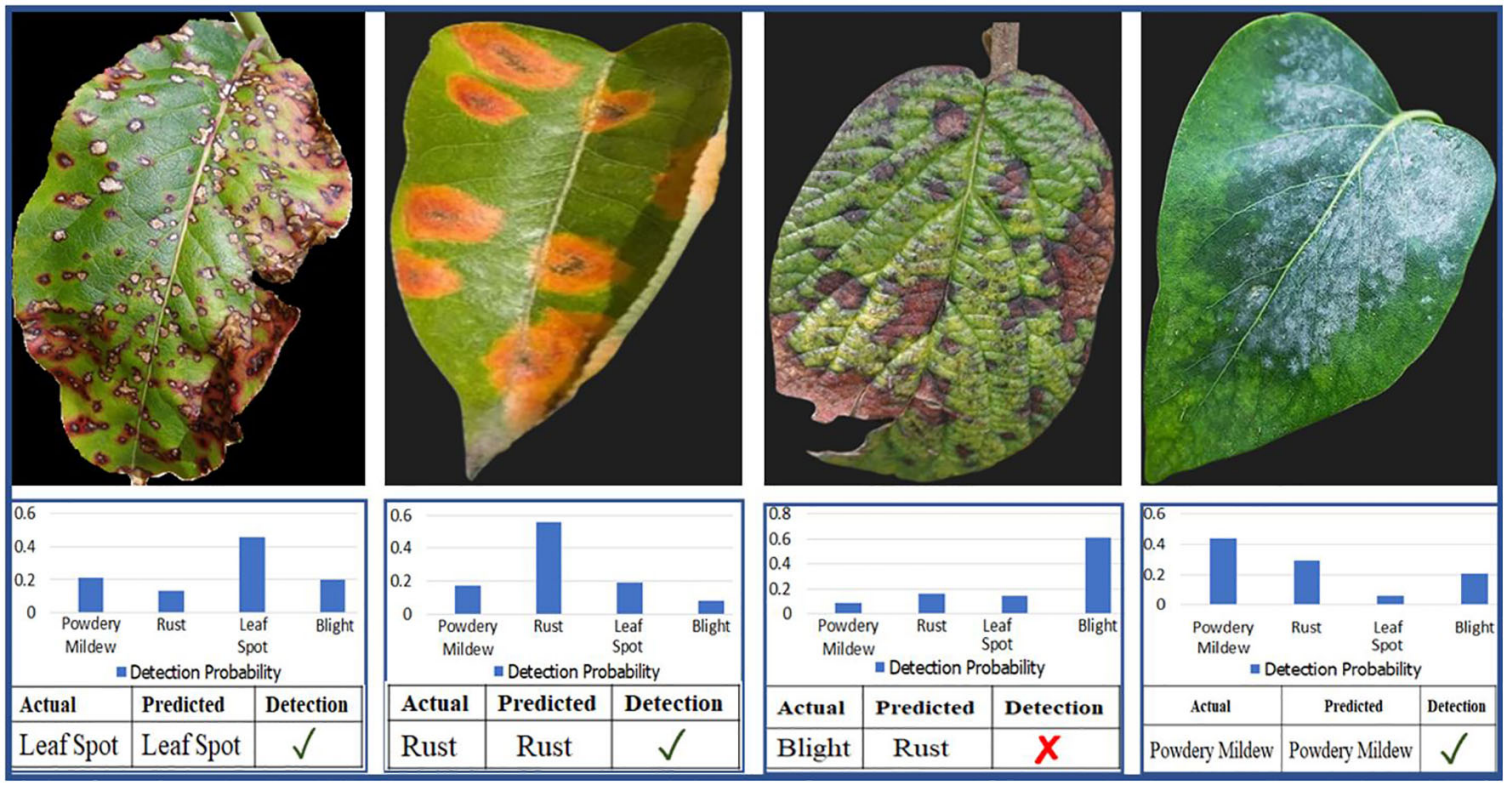
Sample images and corresponding saliency maps for the EG-CNN model.

The **Figure 4** shows the classification probabilities, and plant survival rate as low, moderate or high. CNN was first widely used to detect pests and diseases in plants. There is currently research being done in the categories of image classification after identifying an area of interest (ROI), as well as multi-category classification after identifying an area of interest (ROI).

- An enhanced pre-training model based on transfer learning was proposed by Sakib et al. (Sourav and Wang, 2023). Insect species were classified using three sets of public insect data, which were accurate 96.75 percent, 97.47 percent, and 95.97 percent. Plant and pest diseases were identified using ResNet50 by Lawrence et al. (Ngugi et al., 2021). For identifying the grade of leaf disease using Adam’s optimization approach, 95.61 percent accuracy was achieved by substituting the focus loss function for the traditional cross-entropy loss function.
- As soon as the return on investment has been calculated, the classification process begins. Our method of judging diseases and pests is based on a fixed area within the region of interest (ROI) for the entire acquired image because we often know the ROI beforehand.
- As with the original image classification system, the traditional plant and pest classification network sorting into different categories behaves similarly when categorizing more than two classes, with the network size being 1 (including middle classes. However, methods that employ multiple classes of classification usually start out by identifying lesions and standard samples before sharing characteristic extraction sections to modify or expand the classification branches. By utilizing binary learning between normal and diseased samples and pests, a pre-workout weight parameter can be generated for multi-goal disease and pest classification networks. With a single multi-crop model Kirti et al (Kirti and Rajpal, 2023). identified 17 diseases across five cultures with a CNN architecture that integrates basic metadata. The model can accomplish the following objectives:
  • The goal is to achieve more prosperity and stability through visual characteristics than through a single culture.
  • This culture doesn’t suffer from diseases with similar symptoms to other cultures.
  • Classifies conditional crop diseases based on the context.

### 4.4 Locating lesions with the network

An image is classified using a label by a classification network. There are three common methods: sliding windows, thermal maps, and multitasking learning networks.

- Determine the location of objects based on a sliding window pattern. In general, this is the most intuitive and basic method for determining the location of a lesion. Using CNN classification networks based on the sliding window technique, Chen et al (Chithambarathanu and Jeyakumar, 2023). developed a system to identify and estimate regression of plant disease and pest species positions based on machine learning characteristics. A majority of the 38 common symptoms had been recognized by the field in 50-90 percent of the cases.
- In the temperature chart, regions within the image are ranked based on the hues of their regions. Temperature charts demonstrate how hues indicate intensity of a region. Image analysis and classification are made easier with this tool. In addition to detecting outliers in images, the temperature chart may also be used for data analysis. Darker hues denote greater importance, and darker hues indicate a larger area. Plant diseases and pest infestation are more likely to be identified by darker heat maps. As a result of training CNN to create thermal maps of corn disease images, Kumar and colleagues (Rai and Pahuja, 2024) classified entire images depending on whether they indicated infected leaves or not. The creation of a thermal map of an image takes about two minutes (1.6 GB of memory) and identifying three thermal cards for execution takes less than a second (800MB of memory). Test data show an accuracy of 96.7 percent in experiments. Using the thermal map system, Wiesner-Hanks et al. analyzed maize disease contour zones in 2019. Using the model, lesions as small as millimeters can be accurately detected from drone images with a 99.79 percent accuracy rate, which is an enormous advancement in detecting aerial plant diseases.
- Learning network that performs multiple tasks at once. As long as there is no other capacity in the purely classified network, it will be able only to classify images at the image level. In order to locate plant diseases and pest infestations reliably, the technical network must frequently add a branch. A segmentation network’s branches can be created by learning samples from every pixel in the image. Therefore, the multitasking learning network generates specific lesion segmentation results by using segmentation branches, which reduces the sampling requirements for classification. The deconvolution-guided VGNet model (DGVGNet) was developed by Ren et al (Bouacida et al., 2024). in order to detect plant leaf diseases caused by shadows, occlusions, and light intensity. CNN classifiers have been refocused on actual lesion sites because of deconvolution. The model is robust in occlusions, low light, and other conditions, with an accuracy of 99.19 percent in identifying disease classes, 94.66 percent in segmenting lesions, and 99.19 percent in identifying disease classes.

Many researchers working on plant disease and pest classification (Devi et al., 2023; Liu and Wang, 2021; Li et al., 2021; Turkoglu et al., 2022). As shown in **Table 3**, each sub-method has its own set of advantages and disadvantages.

**Table 3:** Pros and cons of each classification network sub-method are compared.

Method	Advantages	Disadvantages	Application	Implementation Complexity	Resource Requirements
Network Feature Extraction	Detailed lesion feature extraction	Requires additional classifiers, adding complexity	Lesion analysis, feature extraction	Moderate	Moderate
Classification of Original Image	Fundamental framework	Effectiveness depends on lesion percentage in the image	General image classification	Low	Low
ROI-Based Classification	Precise lesion details	Requires additional methods for ROI extraction, adding complexity	Targeted lesion analysis, accurate classification	High	High
Multi-Category Classification	Addresses sample imbalance, considers multiple lesion categories	Requires secondary level training, increasing complexity	Diverse lesion categories, robust classification model	Moderate	Moderate
Sliding Window	Preliminary lesion localization	Optimal window size selection can be time-consuming	Initial lesion detection, broad overview	Moderate	Low
Heatmap	Enhances accuracy in lesion localization	Dependent on classification model accuracy	Detailed lesion localization, improved precision	High	Moderate

### 4.5 Impact of environmental factors on model performance

In real-world agricultural settings, environmental factors such as lighting conditions, background complexity, weather, and seasonal variations play a significant role in the performance of plant disease and pest detection models. These factors can introduce significant variability in image data, which poses challenges for the accuracy and robustness of deep learning models. For instance, variations in natural lighting can cause shadows, overexposure, or underexposure in images, making it difficult for models to distinguish subtle signs of disease or pest infestations. Additionally, the background complexity, such as cluttered foliage or varying soil textures, can interfere with accurate segmentation and detection of target features.

Seasonal changes also contribute to variations in plant appearance, with differences in leaf color, texture, and growth patterns that can affect how diseases manifest on plants. These environmental variations make it crucial for models to generalize well across different conditions. To address these challenges, strategies such as data augmentation, where images are artificially varied through changes in brightness, contrast, and rotation, can help simulate environmental factors and improve model robustness. Transfer learning, which allows models to adapt to new environments using pre-trained models, can also aid in mitigating the impact of environmental variability. By acknowledging and addressing the influence of these factors, the study ensures that the proposed models are more adaptable and reliable in diverse agricultural contexts.

### 4.6 Relevance to small-scale farms and resource-constrained environments

The approaches and models discussed in this study are designed to be applicable and effective in environments with limited resources, such as small-scale farms. These types of farms often face challenges including limited access to high-end computational infrastructure, scarce datasets, and insufficient technical expertise for implementing complex machine learning models. To address these challenges, the study emphasizes the use of lightweight deep learning architectures, such as smaller CNNs and more efficient versions of Generative Adversarial Networks (GANs) and Variational Autoencoders (VAEs), which require less computational power and memory while still delivering accurate results. Moreover, transfer learning plays a crucial role in adapting models to new, data-scarce environments. By fine-tuning pre-trained models on a smaller set of locally collected data, these models can be adapted to the specific conditions of small-scale farms, without the need for large annotated datasets. This significantly reduces the burden of data collection and labeling, which is a major challenge in resource-constrained environments.

Additionally, data augmentation techniques—such as varying lighting, background conditions, and crop types—allow the model to be trained on a diverse range of synthetic images, improving generalization. This is particularly important in small-scale farms where natural environmental variations, such as changes in weather, crop health, and pest behavior, can significantly differ from region to region. The study also discusses the integration of edge computing, where processing is done locally on devices such as smartphones or low-cost IoT sensors, minimizing the need for cloud-based infrastructure. This makes the technology more accessible for small-scale farmers, who may not have access to robust internet connectivity or expensive hardware. By focusing on these strategies—lightweight models, transfer learning, data augmentation, and edge computing—the study ensures that the proposed methods are not only scalable but also practical and accessible for small-scale farms. These approaches provide a pathway for implementing effective plant disease and pest detection systems in resource-constrained environments, ultimately contributing to sustainable agricultural practices.

The adoption of AI-based plant disease detection systems in agriculture introduces both ecological and socio-economic trade-offs, particularly for smallholder farms in developing countries. While these systems offer significant benefits, such as improved crop health monitoring, reduced reliance on chemical pesticides, and increased productivity, they also pose challenges. Ecologically, the deployment of AI technologies may drive the need for energy-intensive infrastructure, such as edge computing devices and cloud-based solutions, potentially increasing carbon footprints. Socio-economically, the high costs of implementation, limited access to digital tools, and lack of technical expertise among smallholder farmers can hinder adoption. Furthermore, the introduction of advanced AI systems may exacerbate existing inequalities, favoring larger farms with better resources. Addressing these trade-offs requires developing cost-effective, energy-efficient, and user-friendly AI systems tailored to the specific needs of smallholder farms, ensuring equitable access and sustainability while minimizing environmental impacts.

### 4.7 Detection network

Computer vision relies heavily on object positioning. As a result, plant diseases and pests can be detected as closely as possible in a traditional sense. Object location and category are its primary aims. It is constantly being developed methods for detecting objects using deep learning. Three types of deep learning-based plant disease and pest infestation detection networks include two-tiered networks like Faster R-CNN (Olgun et al., 2018), one-story networks like SSD (Wang, 2022), and YOLO (Redmon and Bochkovskiy, 2021). The two-tiered network is different in that it must first create a candidate box (proposal) with lesions before it can detect objects. In contrast, one-story networks directly predict lesions based on their features.

#### 4.7.1 Detection of pests and diseases in plants using a two-stage network

Faster R-CNN works in two stages: first obtaining input image maps of functions via the unifying network. Refine the initial detection results after connecting ROI-Carpool to the network, then obtain the lesion position and classification results after refining the initial detection results. Due to this method, plant diseases and pest infestations are detected more accurately by improving the spine structure, anchor ratio, ROI-carpooling, and loss function. The first time Faster R-CNN was used to accurately locate tomato diseases and pest infestations was by Zhang et al. (Zhang et al., 2023). The mAP value rises to 85.98 percent when deep functionality extractors like VGG-Net and ResNet are used. The CNN model’s parameters were modified in 2019 by Ozguven et al. to detect beet spot disease automatically using R-CNN. The total number of qualified and verified images is 155. This system scores 95.48 percent on right-hand rankings. FCM-KM and Faster R-CNN were merged by Sakib et al. (Shovon et al., 2023) for the rapid detection of rice diseases. 96.71 percent of rice explosions, 97.53 percent of bacterial blights, and 98.26 percent of sheath burns could be detected accurately and quickly for 3010 images. A 15.01 frame-per-second detection speed and an accuracy of 81.1 percent were achieved with the proposed model.

#### 4.7.2 One-stage network for disease and pest detection

With this one-step approach, the process of detecting objects becomes significantly quicker and more efficient, making it an attractive option for many applications. Further, compared to traditional two-step object detection approaches, it requires fewer parameters and training time. In computer vision research, SSD and YOLO both come from open sources. Objects can be detected quickly and accurately in images with these tools. Unlike traditional convoluted neural networks, SSD uses a pyramidal network of features to extract functionality and make predictions from various layers. The YOLO method classifies objects in images by using a convolutional neural network. Singh et al. [63] created the PlantDoc dataset to detect plant diseases using this method because it is faster and more accurate than its predecessors. To make model setting detection easier on a mobile CPU, an app based on MobileNets and SSD was developed. Sun et al. [64] showed how to detect corn leaf burn in a complex context using a multi-scale characteristic fusion instance detection system based on a convolutional neural network improved with SSD. Pre-processing data, merging characteristics, sharing characteristics, disease detection, and other measures were all part of the proposed system. The new model has a higher mAP than the SSD model (ranging from 71.80 to 91.83 percent). The SPF of the new model was also increased (from 24 to 28.4), bringing it closer to real-time detection. YOLO approaches end-to-end detection of a single CNN network as a regression problem and uses global knowledge to directly predict the item’s delimitation area and category.

The detection of plant diseases and pests is increasingly based on two-step models that emphasize accuracy. Machine learning is used in these models to identify potential disease outbreaks and pest threats. In addition to predicting future outbreaks, the models can help farmers prepare for and respond to them more effectively. Detailed annotations must be provided in advance in order to determine the location of plant diseases and pests. The model can then produce more accurate predictions since it has a better understanding of the environment. As well as detecting disease outbreaks and pests, these models can detect environmental changes that may be contributing to disease outbreaks. Using their knowledge of diseased and pest-infested areas, researchers and farmers can provide this detail. In order to generate accurate predictions, the model is trained using this data. The type of plant disease and pest determined during training is not always the type that occurs in the field. To a certain extent, a detection network can address “what types of plant diseases and pests are in which areas” if it provides accurate results in a good model differentiation. A classification network, however, can help represent the individuality of plant diseases, while pest categories merely refer to the types of diseases and pests in a given area. Consequently, the classification network can’t perform the same functions as the detection network.

### 4.8 Segmentation network

Segmentation networks detect diseases by identifying lesions and healthy areas using semantic segmentation. As a result of this network, lesions and healthy areas can be accurately classified, and multiple diseases can be detected in one image. Furthermore, it is capable of providing a comprehensive analysis of a patient’s health using this data. Based on the entire area of the lesion, this method calculates the position, rank, surface, contour, and center of the lesion (along with its length, width, and surface area). Next, either benign or malignant lesions are classified by the classification algorithm. In addition to calculating the growth rate of the lesion, the algorithm also calculates the likelihood of it spreading to other parts of the body.

#### 4.8.1 Fully connected network

The semantics of the image are segmented using a complete convolution neural network (FCN). In FCN, the input image features are extracted and encoded by convolution, and the characteristic image is gradually resized by deconvolution or oversampling. Almost every semantic segmentation model uses FCN today. FCNs are classified into three clusters based on their structure: traditional FCNs, U-nets (Zhang and Zhang, 2023), and SegNets (Goncalves et al., 2021).

- FCN as it appeared in its original form. The corn leaf lesion image was deconvoluted to restore its size and resolution, then convolution layers were used to extract the multilayer characteristics. To detect corn leaf lesions, the extracted features were used. A validation and performance evaluation was then conducted on the model. A comparison was made between the results of the automatic detection and those of the manual detection. By segmenting the small area of the lesion, we were able to achieve an accuracy of 95.87 percent compared to the original FCN process.
- In one network, the U-Net acts as both a decoder and a decoder decoder. This algorithm introduces a layer jump relationship, which allows the decoding stage feature map to be merged with that in the coding stage to aid in recovering segmentation information. U-net-based convolutional neural networks were used by Lin et al. (Lin et al., 2019) to segment 50 wild cucumber oidium sheets. In a complex context, the U-net approach segmented the affected area with fewer samples and good accuracy and speed.
- Lastly, SegNet can be considered. In addition, it is a standard encoder-decoder configuration. An unusual aspect of the set-top box oversampling operation is that it uses the most extensive indexes from consolidation. Kerkech and colleagues (Kerkech et al., 2020) presented a segmentation system for unmanned aerial vehicles. Based on four categories of shadows, field vines, stable, and symptomatic images, 488 samples of visible and infrared images were analyzed by SegNet. On vines and leaves, respectively, 92 percent and 87 percent of the proposed method were detected.

#### 4.8.2 Mask R-CNN

Image segmentation methods such as Mask R-CNN are very popular. In this technique, multitasking learning is used for segmentation and detection. With instance segmentation, multiple lesions of the same type can be distinguished from each other and counted. Alternatively, semantic segmentation treats multi-tumors of the same form as a single entity. With the help of an unmanned aerial vehicle (NLB) image, Stewart et al. (DeChant eta al., 2017) were able to divide corn leaf burn injuries into segments using a Mask R-CNN model. The qualified model allows reliable detection and segmentation of a single lesion. The IOU between the actual baseline and the expected lesion was 0.73 at the 0.50 IOU threshold, with a 0.96 average accuracy. As well as identifying diseases and pests with object detection networks, the Mask R-CNN system has been used in several studies. The authors (Wang et al., 2019) used two separate models to detect and segment the infected region, Faster R-CNN and Ask R-CNN, with Faster R-CNN identifying the tomato disease class and Mask R-CNN identifying and segmenting the infected region’s location and shape. Using the proposed model, 11 different types of tomato diseases were easily and reliably classified by location and form. With a detection rate of 99.64 percent, Mask R-CNN detected all tomato disease groups. Compared to classification and identification networks, segmentation provides more information about lesions.

#### 4.8.3 Advantages and practical applications of segmentation networks

Segmentation networks, such as Mask R-CNN and U-Net, are powerful tools in the detection and analysis of plant diseases and pest infestations. These models enable the precise localization and segmentation of affected areas in plant images, making them highly effective in agricultural applications. Mask R-CNN, an extension of Faster R-CNN, is particularly advantageous for instance segmentation, where it not only detects the presence of a disease or pest but also accurately delineates the boundaries of the affected areas. This capability is crucial for quantifying the extent of damage caused by plant diseases or pests. For example, in detecting powdery mildew on crops, Mask R-CNN can separate the fungal growth from the healthy parts of the plant, providing detailed information about the size and location of the infection. A study by Liu et al. (2020) demonstrated that Mask R-CNN could achieve high accuracy in segmenting grapevine leaves affected by downy mildew, offering an effective solution for early detection and targeted treatment.

U-Net, designed for medical image segmentation, has also shown remarkable success in agricultural image analysis due to its encoder-decoder architecture, which allows the model to produce high-resolution output and accurately segment small lesions or infections on plants. U-Net has proven particularly useful in cases where there is a need to segment images with limited labeled data. For instance, Prathusha et al. (2020) applied U-Net to detect tomato leaf curl virus (TLCV), successfully identifying and segmenting infected areas from healthy tissue. This level of precision aids in early disease detection, enabling more targeted interventions and reducing unnecessary pesticide use.

Other examples of segmentation network applications include:

1. Early detection of citrus greening (HLB) disease using U-Net, where it was applied to images of citrus trees to accurately identify infected regions, even when the symptoms were subtle.
2. Rice plant disease detection using Mask R-CNN to segment leaf spot lesions caused by the bacterial blight disease, allowing for precise quantification of the infected area and aiding in the development of better disease management strategies.
3. Weed detection and classification using U-Net to segment crops from weeds in agricultural fields, helping to automate weed management processes and improve crop yields by minimizing herbicide use.

These segmentation networks offer practical applications in precision agriculture, where they enable automated and efficient monitoring of plant health. The detailed segmentation results allow farmers and researchers to accurately assess the severity of plant diseases and pest infestations, leading to more efficient resource management and targeted intervention strategies. Segmentation networks like Mask R-CNN and U-Net provide significant advantages in plant disease and pest detection by offering high precision in segmenting affected areas. Their practical applications, demonstrated through various case studies, show their ability to enhance early detection, improve crop management, and contribute to sustainable farming practices.

## 5 Integration of IoT and edge computing for enhanced plant disease detection

The integration of Internet of Things (IoT) and edge computing technologies has the potential to significantly enhance the process of plant disease detection, offering real-time monitoring and analysis of plant health in agricultural settings. These technologies enable efficient, scalable, and autonomous systems for early disease detection, which is crucial for managing crop health and preventing large-scale outbreaks.

### 5.1 Internet of things in plant disease detection

IoT refers to the network of physical devices, such as sensors and cameras, connected to the internet to collect and exchange data. In agriculture, IoT devices can be deployed throughout a farm to monitor various environmental factors that influence plant health, such as temperature, humidity, soil moisture, and light intensity. These environmental variables are essential for understanding plant disease dynamics, as diseases often thrive under specific conditions. For instance, IoT-enabled sensors can be used to measure soil moisture and temperature, providing valuable insights into conditions that may predispose crops to fungal or bacterial infections. When combined with image data from cameras or drones capturing plant images, IoT devices can provide real-time feedback on plant health, allowing for early detection of diseases such as powdery mildew, downy mildew, or leaf rust. Moreover, IoT networks can support precision agriculture by enabling the automatic collection and transmission of large datasets across the farm. This data can then be processed and analyzed to detect patterns indicative of disease outbreaks. Smart sensors, for example, can continuously monitor plant leaves for visual symptoms of disease, such as spots or lesions, and alert farmers immediately.

### 5.2 Edge computing in plant disease detection

Edge computing complements IoT by enabling local data processing near the source of data generation (i.e., on-site at the farm), reducing the reliance on cloud-based systems and minimizing the delay between data capture and actionable insights. In the context of plant disease detection, edge computing allows for the real-time analysis of images captured by cameras or drones, as well as sensor data, directly on local devices such as smartphones, drones, or field gateways. For example, edge computing allows image processing algorithms, such as CNNs, to run on local devices to detect and classify plant diseases without needing to send all data to a cloud server. This local processing ensures quicker results and more efficient disease detection, even in remote areas with limited or unreliable internet connectivity. By reducing latency and providing immediate feedback, edge computing enhances the timeliness of interventions, allowing farmers to act quickly before diseases spread further. In addition, by offloading intensive computations to local edge devices, it reduces the bandwidth and data transfer costs associated with cloud-based systems. This is particularly important for large-scale farms or areas with limited internet infrastructure. Below are some examples discussed for the application of IoT and edge computing in the agricultural domain.

1. IoT-based systems, such as Plantix, use sensors and mobile apps to detect plant diseases by analyzing images captured by farmers. The app processes the images and environmental data to provide real-time disease diagnosis and recommendations for treatment. By integrating IoT-enabled sensors with image recognition technologies, it offers a more comprehensive and accurate disease detection solution.
2. John Deere, a leader in agricultural machinery, uses edge computing in its smart farming equipment to detect plant diseases and pests in real-time. Their See & Spray technology uses high-resolution cameras and on-board computing power to identify weeds, pests, and diseases while simultaneously applying targeted treatments, reducing resource waste and ensuring efficient disease management.
3. IoT-enabled irrigation systems can help reduce the risk of plant diseases caused by overwatering or under-watering. These systems use soil moisture sensors to provide real-time data to farmers, who can adjust irrigation schedules accordingly. In combination with edge computing, these systems can monitor environmental conditions and plant health simultaneously, enhancing disease prevention.

## 6 Analyzing the dataset and comparing its performance

In this section, we review plant disease and pest datasets and compare and analyze related deep learning models.

### 6.1 Datasets for plant diseases and pests detection

In order to detect plant diseases and pests, scientists use datasets. Plant and pest diseases aren’t detected by a comprehensive and unified dataset, such as ImageNet, PASCALVOC 2007/2012, or COCO. To collect plant disease and pest data, self-collection, networking, and public databases can all be used. Various methods are frequently used to collect image data sets, including unmanned aerial remote sensing, ground camera imagery, video capture with the Internet of Things, aerial photography using a camera, hyperspectral imaging, near-infrared spectrometers, and others. A widely used digital library, PlantVillage, is a popular resource for public datasets. The natural world, however, collects more realistic data on plant diseases and pests. It can be difficult to compare field-collected photographs across diseases, artifacts, and detection scenarios systematically, even though several researchers distribute field-collected photographs. Various datasets related to detecting plant and pest diseases are provided in this section, according to existing research. The results of the study are summarized in **Table 4**.

**Table 4:** Plant disease and pest detection from benchmark datasets.

Dataset Name	Species	Collection Environment	Link	Number of Images	Classes	Background
PlantVillage-Dataset	14 crop varieties, 26 diseases	Detached leaves on a plain background	GitHub	50,000	26	No
Rice Leaf Diseases	Rice	Captured with a white background in direct sunlight	UCI Archive	Not specified	3	No
Plant Disease Symptoms Image Database (PDDB)	21 plant species	Field	Embrapa	2,326	171	No
New Plant Diseases Dataset (Augmented)	Various crop leaves	Detached leaves on a plain background	Kaggle	87,000	38	No
PlantVillage Dataset	Not specified	Network	GitHub	Not specified	39	Yes
Northern Leaf Blight (NLB) Lesions	Not specified	Field	OSF	105,705	Not specified	No
Insect Pests Database	Rice, maize, soybean, sugarcane, cotton	Field	NBAGR	Not specified	40	No
High-Quality Crop Images	Rice, wheat, maize	Not specified	Website	Not specified	Not specified	No
PlantDoc Dataset	13 plant species, 17 diseases	Field	GitHub - Object Detection	2,598	17	No
Maize Dataset for NLB	Maize	Field	Bisque	Not specified	Not specified	No
Apple Leaf Disease	Apple	Field	Kaggle	3,651	Not specified	No
IP102 Insect Pest Recognition Database	Various insect pests	Field	GitHub	75,000	102	No
Tomato Pests Database	Tomato	Network	Mendeley	8	Not specified	No

## 7 Evaluation indices

Evaluation indices will vary based on the study focus. Metrics used to evaluate performance include precision, recall, and harmonic mean F1. Precision and recall are defined as:


(1) $$Precision=\frac{TP}{TP+FP}\;*\;100$$

(2) $$Recall=\frac{TP}{TP+FN}\;*\;100$$

It is estimated that TP (True Positive) represents the number of lesions correctly detected in Formulas 1 and 2. Between one and zero lesions are estimated to be incorrectly detected by the algorithm. False Positives (FP) indicate that the algorithm detected lesions incorrectly. In FN codes, the number of unidentified lesions ranges from 0 to 1. The method of determining detection accuracy known as mAP is widely used. To begin, calculate the average accuracy of each dataset segment:


(3) $$P_{average}=\sum_{j=1}^{N(Class)}Precision(j)\;*\;Recall(j)\;*\;100$$

The formula above has N (class) representing the total number of categories, Precision (j) representing precision and recall for class j, and Recall (j) representing recall for class j, respectively. Accordingly, each category’s mAP is calculated as follows:


(4) $$mAP=\frac{P_{average}}{N(Class)}$$

An algorithm’s recognition accuracy increases as its mAP value increases; the algorithm’s recognition accuracy decreases as its mAP value decreases. In addition to the F1 score, the accuracy of the model is also evaluated. Model precision and recall are both considered in the F1 score. The following is the formula:


(5) $$F1=2\;*\;\frac{Precision\;*\;Recall}{Precision+Recall}\;*\;100$$

FPS measures recognition speed. With increasing frames per second, the algorithm recognizes more objects. Alternatively, recognition speed decreases with a reduction in frames per second.

### 7.1 Performance comparison of existing algorithms

A complete classification, diagnosis, and segmentation of the samples has been accomplished as well as more complex tasks such as determining the degree of infection. Based on unique data sets, the most advanced methods for detecting pest infestations and plant diseases are used. It is still difficult to compare all algorithms consistently due to the lack of comprehensive, publicly accessible datasets. In recent years, some popular algorithms have steadily improved their performance on various datasets, with improved mAP, F1 score, and FPS. Despite significant advances in previous research, there is still a significant gap between the sophistication of infectious disease and pest images used in current research and real-time disease detection using mobile devices in the field. Data sets that are larger, more complex, and experimental will be required for future research.

## 8 Challenges

### 8.1 Small dataset size problem

Plant diseases and pests can now be identified using deep-learning approaches in specialized agricultural applications. Insufficient samples of agricultural plants have been collected for disease and pest research. It is difficult to mark self-collected datasets compared to open standard libraries. While ImageNet datasets contain over 14 million samples, detecting plant and pest diseases remains a challenge because of the small sample size. A few or dozens of training data are available for detecting diseases and plant pests due to low prevalence and high acquisition costs. Currently, small samples are approached in three ways.

### 8.2 Data amplification, synthesis, and generation

As part of the training process for deep learning models, data amplification is required. A well-designed data amplification technique will greatly aid the detection of plant diseases and pests. Mirroring, rotating, flipping, deforming, filtering, and contrast adjustment can produce more samples. Small data sets will also be enriched by GAN (Nazki et al., 2020) and Automatic Variational Encoder (VAE) (Zilvan et al., 2019). Emerging datasets, including synthetic data generated through GANs, play a pivotal role in addressing data scarcity and improving the robustness of deep learning models for plant disease and pest detection. GANs can create realistic synthetic images that augment existing datasets, enabling models to generalize better across diverse scenarios and conditions. This approach is particularly beneficial in cases where collecting real-world data is challenging due to seasonal limitations, rare disease occurrences, or resource constraints. Moreover, synthetic data generation helps balance datasets by addressing class imbalances, such as underrepresented diseases or pests, thereby enhancing model performance. By leveraging GANs to create high-quality, diverse datasets, researchers can train more robust and scalable models, making AI-based solutions more effective and accessible for real-world agricultural applications.

### 8.3 Fine-tuned and transfer learning

Transfer Learning (TL) involves transferring information from large, generic datasets to sparsely represented areas. In transfer learning, a learning dataset can be used as a starting point when creating a model for newly collected, unannotated samples. Natural light was used to photograph contaminated potatoes of various shapes, sizes, and hues. The VGG network was then used to identify the potatoes. In addition to new learning, network transfer training was also beneficial. In their comparison of conventional networks, Too et al. (2019) used fine and contrasting settings. Increasing the number of iterations improved the accuracy of Dense-Nets. They achieved an average accuracy of 92.00 percent using transfer learning and adjustment to properly categorize rice disease images in a complex context, demonstrating that transfer learning is more effective than traditional training.

### 8.4 An appropriate network structure

A network structure can significantly reduce sample requirements. Using three color components, Zhang et al. (2019) developed a convolution neural network model for recognizing plant leaf diseases. For each channel of TCCNN, there are three-color RGB leaf disease images. An improved CNN method was proposed by Liu et al. (2017) for the detection of disease in vine leaves. Deep separable convolution was used instead of regular convolution to avoid overadjustment and reduce parameters. To improve the extraction of multi-scale characteristics for vine leaf lesions of different sizes, the original structure was added to the model. Traditional ResNet and GoogleLeNet structures have slower convergence speeds and lower training accuracy.

### 8.5 Identifying small lesions in an early stage

#### 8.5.1 Early detection of small lesions

In order to maximize yields, plant diseases must be detected early (Bouacida et al., 2024). Small-scale artifacts are often overlooked in the deep characteristic extraction network due to the size of the lesion object. Also, background noise on the collected images can result in false detection because of the large-scale complexity of the background, especially on low-resolution images. Small object detection is examined in light of the scarcity of existing algorithms. In order to improve the detection efficiency of small targets, several techniques have been proposed, such as the use of an attention mechanism. By using the attention system, resources can be allocated more rationally. Attention is primarily responsible for locating a region of interest and discarding irrelevant information quickly. Using the factory village dataset, Karthik et al. (2020) tested a residual network attention mechanism with 98 percent accuracy. In this method, a protruding image is obtained, the object is isolated from its context, the characteristic image is manipulated, and the original characteristic image is combined with the characteristic image. A new fusion function can be created by the Attention Mechanism module using the Softmax function in order to reduce noise. By using attention mechanisms, we will be able to select information and allocate resources more accurately in future studies on the early detection of plant diseases and pests. A residual network attention mechanism was tested with 98 percent accuracy using the factory village dataset by Karthik et al. (2020).

#### 8.5.2 Fine-grained identification

There are a lot of variations; for example, different plant diseases and pests can appear slightly different from one another. Irregular lighting, dense occlusion, blurred equipment weaving, or other interference can lead to differences in samples of the same disease or pest. A complex situation makes it difficult to identify plant diseases and pests (Wang, 2022). There is also some blurring of class that makes objects from different classes look the same. There are similarities between subclasses in biological morphology and lifestyle that pose the problem of fine recognition of “interclass similarity.” Despite similar symptoms, Barbedo, 2019 says that plant pathologists cannot distinguish them. The detection of plant and pest diseases becomes more difficult when the story interacts with other objects of interest. Some publications overlook this issue since photographs are taken under controlled conditions.

### 8.6 Real-world feasibility, scalability, and challenges of deployment

While the proposed deep learning-based techniques for plant disease detection show significant promise, their deployment in real-world agricultural settings presents several challenges that need to be carefully considered. The feasibility of implementing these techniques depends on several factors, including the availability of high-quality data, suitable hardware, and the technical expertise required for deployment. In agricultural environments, deploying models such as CNNs or GANs requires access to robust computational resources, especially when dealing with large datasets. Moreover, real-time processing capabilities are crucial to make decisions promptly in large-scale farms. Therefore, employing edge computing and IoT solutions can significantly aid in processing data locally, reducing latency, and improving overall system efficiency.

Scalability is another key consideration, particularly when moving from small-scale to large-scale farms. The proposed models should be adaptable to different farm sizes, crop types, and environmental conditions. For large farms, the models must be capable of handling high volumes of data collected from various sensors and cameras. Additionally, scaling the models to accommodate diverse environmental factors such as lighting conditions, plant species, and pest varieties may require fine-tuning or transfer learning strategies. Several challenges need to be addressed for the effective deployment of plant disease detection systems. The first challenge is data availability, particularly in regions with limited access to high-quality, labeled datasets. Variability in environmental factors (e.g., weather conditions, light intensity, and background noise) can also impact model accuracy. Furthermore, many small-scale farms may lack the necessary infrastructure, such as high-speed internet and advanced hardware, to deploy complex deep learning models. These limitations make it essential to develop lightweight, cost-effective solutions that can be deployed without significant resources.

To overcome these challenges, future research should focus on developing scalable models that can be easily adapted to different agricultural contexts. Additionally, incorporating IoT and edge computing technologies can help address data processing and real-time decision-making requirements, making the detection system more practical for deployment in resource-constrained environments.

### 8.7 Detection performance under the influence of illumination and occlusion

#### 8.7.1 Lighting problems

The use of indoor lightboxes has been used in the past to capture diseases and pests on plants [105]. It can simplify image processing by eliminating the impact of external light, but the result is a very different image captured in natural light. The dynamic range of natural light is small, which makes it easy for a camera’s dynamic light source range to become out of date when it is used with natural light. Moreover, due to differences in angle of view and distance, the appearance of plant and pest diseases changes significantly during image processing.

#### 8.7.2 Occlusion problem

There is currently a lack of efforts by scientists to identify plant diseases and pests across various ecosystems. Whenever they are dealing with a situation, they only pay attention to that situation. Often, they do not consider the occlusion problem when collecting images of areas of interest. Consequently, recognition accuracy and practicality cannot be maximized under occlusion. The occlusion of the blade, the occlusion of the branch, and the occlusion of light due to external lighting are all common causes of occlusion in virtual natural environments. Diseases and pests are difficult to identify due to occlusion and a lack of characteristics. Recognition algorithms are affected differently by different degrees of occlusion, resulting in false or missed detections. Researchers have found it more difficult to identify plant diseases and pests in extreme conditions as deep learning algorithms have improved under limited conditions in recent years. Plant and pest disease identification has made significant progress, providing a solid foundation for real-world applications. In addition to designing lightweight network architecture, the basic framework needs to be improved for creativity and optimization. In contrast, occlusion occurs unexpectedly and is difficult to predict. While retaining detection accuracy, GAN exploration should be improved and model formation complexity reduced. When it comes to posture changes and chaotic environments, THE GAN has many advantages. Although its architecture is still in its infancy, it is easily planted during training, resulting in intractable model problems. Improved exploration of network output will help us measure the model’s effectiveness.

#### 8.7.3 Detection speed problem

There is a substantial difference between deep learning algorithms and traditional ones in terms of computational requirements, but they produce better results. As a result of the model’s familiarity with the image’s characteristics, high detection accuracy results in slow detection speeds and inability to meet real-time requirements. Usually, reducing measurements is necessary to ensure detection speed. In some cases, this can result in inaccurate or missed identifications as a result of a lack of planning. This necessitates developing a quick and accurate threat detection algorithm. A deep learning method for detecting diseases and plant pests in agriculture relies on data labeling, model formation, and model inference. In real-time agricultural applications, model inference is becoming more popular. Most methods for detecting plant and pest diseases emphasize precise identification. The model inference is rarely examined for its reliability. Tests have been conducted on several models. In comparison to VGG and MobileNet, the reduced MobileNet had a classification accuracy of 98.34 percent with settings 29 times lower. Using mobile devices with limited resources in real-time to diagnose crop diseases demonstrates a good balance between time and accuracy.

#### 8.7.4 Identifying and addressing limitations to improve practical applicability

While the models and approaches discussed in this study show considerable promise for plant disease and pest detection, certain limitations may restrict their practical applicability in real-world agricultural settings. One significant challenge is the variability of environmental conditions, such as lighting, weather, and background complexity, which can significantly impact model accuracy and reliability. Addressing these challenges through techniques like data augmentation, which simulates different environmental conditions, and transfer learning, which allows models to adapt to new environments, is essential for improving their robustness and generalization across diverse settings. Another limitation is the dependency on large, labeled datasets for training deep learning models. In resource-constrained environments, such as small-scale farms, the collection and labeling of data can be prohibitively expensive and time-consuming. To mitigate this, the study emphasizes the use of synthetic data generated through techniques like GANs and VAEs, which can augment existing datasets and reduce the need for extensive data collection efforts.

Furthermore, model complexity and computational requirements can be barriers to deployment in environments with limited resources. To address this, the article explores the development of lightweight models that can be deployed on edge devices, minimizing the need for high-end infrastructure and enabling on-site detection. By identifying these key limitations—environmental variability, data scarcity, and computational constraints—and proposing targeted solutions, this study aims to enhance the practical applicability and effectiveness of deep learning models for plant disease and pest detection, particularly in real-world agricultural settings.

## 9 Future directions

In contrast to traditional image processing techniques, which intervene in the detection of plant and pest diseases in stages, deep learning-based methods integrate them into an end-to-end extraction of characteristics. This has a lot of potential. Despite rapid advances in plant and pest disease detection technology, it has moved from academic research to agricultural application. The mature application still requires a great deal of work, and several issues must be resolved before it can be used in the real world.

### 9.1 Plant diseases and pests detection dataset

In recent years, deep learning has made it easier to detect plant diseases and pests. It lays the foundation for identifying complex diseases and pests by improving and expanding image recognition algorithms. Researchers primarily collect photographs of plant diseases and pests in the laboratory and use these photographs as the basis for their research findings. In contrast, previous research collected sample images primarily from identifying disease spots, insects, and insect pests and leaves. Growth in plants occurs cyclically, consistently, seasonally, and regionally. As crops develop, diseases and pests also change. Plant species are pictured differently from one place to another. As a result, most current research findings do not apply universally. Electromagnetic waves produce large amounts of data outside the visible range, however. Due to this, multispectral, near-infrared, and visible light data must be combined to obtain plant disease statistics. Assuring the completeness and accuracy of the data set can also help improve the algorithm’s performance.

### 9.2 Early recognition of plant diseases and pests

In the absence of obvious symptoms, it can be difficult to detect plant and pest diseases early, whether by visual observation or computerized analysis. Research and demand are more important for early diagnosis due to their benefits in preventing and controlling disease and pest spread and growth. On a cloudy day, however, pre-processing is more difficult and the recognition effect is reduced. Taking photos in daylight is best, but on cloudy days, image quality is best. The early stages of plant diseases and pests can also make it difficult to interpret even high-resolution images. Temperature and humidity data, as well as weather data, must be combined to recognize and predict diseases and pests. Plant diseases and pests are rarely diagnosed early using current research literature.

### 9.3 Network training and learning

When plant diseases and pests are detected manually, only reliable data are available (positive samples). The collection of labeled data sets is difficult, however, because most existing approaches use supervised learning and use many samples. A study of unsupervised learning is necessary. Due to the black-box nature of deep learning, it is necessary to label several training samples for end-to-end learning. Additionally, prior knowledge of brain-inspired computation is also useful in guiding network training and learning. While deep models require more memory and testing time, they are incompatible with mobile platforms with limited resources. A fast-paced model needs to have less complexity and be accurate without losing speed.

### 9.4 Interdisciplinary research

We will develop a field diagnostic model based on scientific evidence and theories such as plants’ agronomic defenses to enhance crop growth. In this way, pests and plant diseases will be detected faster and more accurately. Identifying disease and pest occurrence mechanisms and establishing an experimental framework will be critical in the future, as well as incorporating crop growth laws, environmental factors, and other factors into realistic application research.

#### 9.4.1 Multimodal data fusion

One promising avenue for advancing plant disease detection is the integration of multimodal data, combining imagery from various sources (e.g., visible light, thermal, and hyperspectral images) with environmental data from IoT sensors. By fusing data from different modalities, it is possible to gain a more comprehensive understanding of plant health. This approach can overcome limitations such as variations in lighting conditions, and different types of diseases that manifest in various environmental contexts. Effective data fusion can lead to more accurate detection, reduced false positives, and enhanced decision-making in real-world settings.

#### 9.4.2 Scalable models for diverse environments

Another important future direction is the development of scalable models that can be effectively deployed across a wide range of agricultural environments, from smallholder farms to large-scale industrial operations. This includes creating lightweight and adaptable models that can handle the variability in environmental conditions, crop types, and disease profiles. Leveraging techniques like transfer learning and domain adaptation can ensure that models trained in one setting are adaptable to others with minimal retraining. This scalability can bridge the gap between research and real-world deployment, making advanced plant disease detection accessible to farmers across diverse regions.

#### 9.4.3 Edge AI for real-time monitoring

With the continued expansion of edge computing capabilities, deploying AI models directly at the point of data collection—such as on drones, IoT devices, or mobile platforms—will enable real-time disease detection in remote or resource-constrained environments. Future research should focus on enhancing the efficiency and accuracy of edge AI models, ensuring they are lightweight and capable of running in low-resource settings. This will allow farmers to make informed decisions quickly, without the need for constant connectivity to cloud servers.

#### 9.4.5 Automated decision support systems

The future of plant disease management will involve developing intelligent decision support systems that can not only detect diseases but also recommend specific, actionable solutions. These systems should integrate machine learning with expert knowledge and agricultural best practices, offering real-time insights and treatment recommendations tailored to the specific conditions of a farm. By combining disease detection with pest control strategies and optimal treatment schedules, these systems can automate the entire decision-making process, making it easier for farmers to adopt precision agriculture methods.

## 10 Conclusion

The integration of deep learning and computer vision in the detection and analysis of plant diseases and pest infestations has demonstrated significant advancements and potential. Techniques such as sliding windows, thermal maps, multitasking learning networks, and various segmentation methods have enhanced the accuracy and efficiency of identifying and classifying plant health issues. The application of two-stage networks like Faster R-CNN and one-stage networks like SSD and YOLO has particularly improved the speed and precision of detection, making these techniques invaluable for modern agricultural practices. Despite these advancements, challenges remain, particularly in handling diverse environmental conditions and varying disease manifestations. The complexity of accurately segmenting lesions and distinguishing between similar disease symptoms necessitates continuous improvement and innovation in these technologies. Additionally, the high resource requirements and computational demands of some methods pose barriers to widespread adoption, especially in resource-limited settings. Future directions in this field should focus on the following areas related to Enhanced Model Robustness, Integration with IoT and Edge Computing, Scalable and Efficient Models, Multimodal Data Fusion, Automated Annotation and Data Augmentation, and Collaborative Platforms.

## References

[B1] Ahmed S. F. Md S. B.A. Maruf H. Mahtabin R. R. Taoseef I. Nazifa. R.. (2023). Deep learning modelling techniques: current progress, applications, advantages, and challenges. Artificial Intelligence Review (Springer Netherlands) 56, no. 11, 13521–13617. doi: 10.1007/s10462-023-10466-8

[B2] Alzubaidi L. Zhang J. Humaidi A.J. Al-Dujaili A. Duan Y. Al-Shamma O.. (2021). Review of deep learning: concepts, CNN architectures, challenges, applications, future directions. J. Big Data (Springer Nature Germany: Springer International Publishing) 8, 1–74. doi: 10.1186/s40537-021-00444-8 33816053 PMC8010506

[B3] Barbedo J. G. A. (2019). Plant disease identification from individual lesions and spots using deep learning. Biosyst. Eng. 180, 96–107. doi: 10.1016/j.biosystemseng.2019.02.002

[B4] Bouacida I. Farou B. Djakhdjakha L. Seridi H. Kurulay M. (2024). Innovative deep learning approach for cross-crop plant disease detection: A generalized method for identifying unhealthy leaves. Inf. Process. Agric. 1, 1–14. doi: 10.1016/j.inpa.2024.03.002

[B5] Chan K. Y. Abu-Salih B. Qaddoura R. Ala'M A. Z. Palade V. Pham D. S.. (2023). Deep neural networks in the cloud: Review, applications, challenges and research directions. Neurocomputing. 545, 126327. doi: 10.1016/j.neucom.2023.126327

[B6] Chithambarathanu M. Jeyakumar M. K. (2023). Survey on crop pest detection using deep learning and machine learning approaches. Multimed. Tools Appl. 82, 42277–42310. doi: 10.1007/s11042-023-15221-3 PMC10088765 37362671

[B7] C. Science Kingdom U. Neural C. Learning D. C. Engineering (2021). Art classification with pytorch using transfer learning 1–5. doi: 10.1109/ICSCAN53069.2021.9526457

[B8] DeChant C. Wiesner-Hanks T. Chen S. Stewart E. L. Yosinski J. Gore M. A.. (2017). Automated identification of northern leaf blight-infected maize plants from field imagery using deep learning. Phytopathology 107 (11), 1426–1432. doi: 10.1094/PHYTO-11-16-0417-R 28653579

[B9] Devi R. S. Kumar V. R. Sivakumar P. (2023). EfficientNetV2 model for plant disease classification and pest recognition. Comput. Syst. Sci. Eng. 45 (2). doi: 10.32604/csse.2023.032231

[B10] Diana Andrushia A. Mary Neebha T. Trephena Patricia A. Umadevi S. Anand N. Varshney A. (2023). Image-based disease classification in grape leaves using convolutional capsule network. Soft Comput. 27, 1457–1470. doi: 10.1007/s00500-022-07446-5

[B11] Goncalves J. P. Pinto F. A. Queiroz D. M. Villar F. M. Barbedo J. G. Del Ponte E. M. (2021). Deep learning architectures for semantic segmentation and automatic estimation of severity of foliar symptoms caused by diseases or pests. Biosyst. Eng. 210, 129–142. doi: 10.1016/j.biosystemseng.2021.08.011

[B12] Hurtado J. Salvati D. Semola R. Bosio M. LoMonaco V. (2023). Continual learning for predictive maintenance: Overview and challenges. Intell. Syst. Appl. 19, 200251. doi: 10.1016/j.iswa.2023.200251

[B13] Joseph D. S. Pawar P. M. Pramanik R. (2023). Intelligent plant disease diagnosis using convolutional neural network: a review. Multimed. Tools Appl. 82, 21415–21481. doi: 10.1007/s11042-022-14004-6

[B14] Joshua S. V. Priyadharson A. S. M. Kannadasan R. Khan A. A. Lawanont W. Khan F. A.. (2022). Crop yield prediction using machine learning approaches on a wide spectrum. Comput. Mater. Contin. 72, 5663–5679. doi: 10.32604/cmc.2022.027178

[B15] Kabir M. F. Chen T. Ludwig S. A. (2023). A performance analysis of dimensionality reduction algorithms in machine learning models for cancer prediction. Healthc. Anal. 3, 100125. doi: 10.1016/j.health.2022.100125

[B16] Karthik R. Hariharan M. Anand S. Mathikshara P. Johnson A. Menaka R. (2020). Attention embedded residual CNN for disease detection in tomato leaves. Appl. Soft Computing 86, 105933. doi: 10.1016/j.asoc.2019.105933

[B17] Karvelis P. Mazzei D. Biviano M. Stylios C. (2020). Portweather: A lightweight onboard solution for real-time weather prediction. Sensors (Switzerland) 20, 1–21. doi: 10.3390/s20113181 PMC7309033 32503318

[B18] Kaya Y. Gürsoy E. (2023). A novel multi-head CNN design to identify plant diseases using the fusion of RGB images. Ecol. Inform. 75, 101998. doi: 10.1016/j.ecoinf.2023.101998

[B19] Kerkech M. Hafiane A. Canals R. (2020). Vine disease detection in UAV multispectral images using optimized image registration and deep learning segmentation approach. Comput. Electron. Agric. 174, 105446. doi: 10.1016/j.compag.2020.105446

[B20] Khan A. A. Faheem M. Bashir R. N. Wechtaisong C. Abbas M. Z. (2022). Internet of things (IoT) assisted context aware fertilizer recommendation. IEEE Access 10, 129505–129519. doi: 10.1109/ACCESS.2022.3228160

[B21] Khare S. K. Blanes-Vidal V. Nadimi E. S. Acharya U. R. (2024). Emotion recognition and artificial intelligence: A systematic review (2014–2023) and research recommendations. Inf. Fusion 102, 102019. doi: 10.1016/j.inffus.2023.102019

[B22] Kiobia D. O. Mwitta C. J. Fue K. G. Schmidt J. M. Riley D. G. Rains G. C. (2023). A review of successes and impeding challenges of IoT-based insect pest detection systems for estimating agroecosystem health and productivity of cotton. Sensors 23, 4127. doi: 10.3390/s23084127 37112469 PMC10146184

[B23] Kirti Rajpal N. (2023). A multi-crop disease identification approach based on residual attention learning. J. Intell. Syst. 32, 20220248. doi: 10.1515/jisys-2022-0248

[B24] Li X. Wang Y. Chen X. (2022). A comparative study of convolutional neural networks for driver activity recognition in diverse lighting conditions. IEEE Trans. Intell. Transp. Syst. 23, 13245–13257. doi: 10.1109/ACCESS.2024.3489220

[B25] Li L. Zhang S. Wang B. (2021). Plant disease detection and classification by deep learninga review Vol. 9 (IEEE Access), 56683–56698. doi: 10.1109/ACCESS.2021.3069646

[B26] Lin K. Gong L. Huang Y. Liu C. Pan J. (2019). Deep learning-based segmentation and quantification of cucumber powdery mildew using convolutional neural network. Front. Plant Sci. 10. doi: 10.3389/fpls.2019.00155 PMC6413718 30891048

[B27] Liu W. Wang Z. Liu X. Zeng N. Liu Y. Alsaadi F. E. (2017). Neurocomputing A survey of deep neural network architectures and their applications ☆. Neurocomputing 234, 11–26. doi: 10.1016/j.neucom.2016.12.038

[B28] Liu B. Ding Z. Tian L. He D. Li S. Wang H. (2020). Grape leaf disease identification using improved deep convolutional neural networks. Front. Plant Sci. 11. doi: 10.3389/fpls.2020.01082 PMC7373759 32760419

[B29] Liu J. Wang X. (2021). Plant diseases and pests detection based on deep learning: a review. Plant Methods 17, 1–18. doi: 10.1186/s13007-021-00722-9 33627131 PMC7903739

[B30] Liu W. Wang Z. Liu X. Zeng N. Liu Y. Alsaadi F. E. (2017). A survey of deep neural network architectures and their applications. Neurocomputing 234, 11–26. doi: 10.1016/j.neucom.2016.12.038

[B31] Ma P. Li C. Rahaman M. M. Yao Y. Zhang J. Zou S.. (2023). A state-of-the-art survey of object detection techniques in microorganism image analysis: from classical methods to deep learning approaches. Artif. Intell. Rev. 56 (2), 1627–1698. doi: 10.1007/s10462-022-10209-1 35693000 PMC9170564

[B32] Maity A. Paul D. Lamichaney A. Sarkar A. Babbar N. Mandal N.. (2023). Climate change impacts on seed production and quality: current knowledge, implications, and mitigation strategies. Seed Sci. Technol. 51, 7–38. doi: 10.15258/sst.2023.51.1.07

[B33] Mehmood I. Sajjad M. Muhammad K. Shah S. I. A. Sangaiah A. K. Shoaib M.. (2019). An efficient computerized decision support system for the analysis and 3D visualization of brain tumor. Multimed. Tools Appl. 78 (1), 12723–26. doi: 10.1007/s11042-018-6027-0

[B34] Mendoza-Bernal J. González-Vidal A. Skarmeta A. F. (2024). A Convolutional Neural Network approach for image-based anomaly detection in smart agriculture. Expert Syst. Appl. 247, 123210–12748. doi: 10.1016/j.eswa.2024.123210

[B35] Moustafa N. Turnbull B. Choo K. K. R. (2019). An ensemble intrusion detection technique based on proposed statistical flow features for protecting network traffic of internet of things. IEEE Internet Things J. 6, 4815–4830. doi: 10.1109/JIoT.6488907

[B36] Nazki H. Yoon S. Fuentes A. Park D. S. (2020). Unsupervised image translation using adversarial networks for improved plant disease recognition. Comput. Electron. Agric. 168, 105117. doi: 10.1016/j.compag.2019.105117

[B37] Ngugi L. C. Abdelwahab M. Abo-Zahhad M. (2021). A new approach to learning and recognizing leaf diseases from individual lesions using convolutional neural networks. Inf. Process. Agric. 1, 11–27. doi: 10.1016/j.inpa.2021.10.004

[B38] Olgun M. C. Baytar Z. Akpolat K. M. Koray Sahingoz O. (2018). “Autonomous vehicle control for lane and vehicle tracking by using deep learning via vision,” in 2018 6th Int. Conf. Control Eng. Inf. Technol. CEIT. (Hammamet-Tunisia: IEEE), 1–7.

[B39] Parashar N. Johri P. Khan A. A. Gaur N. Kadry S. (2024). An integrated analysis of yield prediction models: A comprehensive review of advancements and challenges. Comput. Mater. Contin. 80, 389–425. doi: 10.32604/cmc.2024.050240

[B40] Prathusha P. Murthy K. S. Srinivas K. (2020). “Plant disease detection using machine learning algorithms,” in Advances in Computational and Bio-Engineering: Proceeding of the International Conference on Computational and Bio Engineering, 2019, Vol. 2. 213–220 (Springer International Publishing). doi: 10.1007/978-3-030-46943-6_25

[B41] Rai C. K. Pahuja R. (2024). Northern maize leaf blight disease detection and segmentation using deep convolution neural networks. Multimed. Tools Appl. 83, 19415–19432. doi: 10.1007/s11042-023-16398-3

[B42] Redmon J. Bochkovskiy A. (2021). YOLOv7-tiny: compact YOLOv4 variant. Pattern Recognit. Lett. 3, 82–93. doi: 10.48550/arXiv.2004.10934

[B43] Sembiring A. Away Y. Arnia F. Muharar R. (2023). “The performance of various concise convolutional neural network configurations in classifying tomato diseases based on leaf images,” in Proceeding of the 3rd International Conference on Electronics, Biomedical Engineering, and Health Informatics: ICEBEHI 2022, Surabaya, Indonesia (Springer), 5–6 October. 373–389.

[B44] Shah S. M. Zhaoyun S. Zaman K. Hussain A. Shoaib M. Lili P. (2022). A driver gaze estimation method based on deep learning. Sensors 22, 1–22. doi: 10.3390/s22103959 PMC9142909 35632365

[B45] Shoaib M. Shah B. Ei-Sappagh S. Ali A. Ullah A. Alenezi F.. (2023). An advanced deep learning models-based plant disease detection: A review of recent research. Front. Plant Sci. 14, 1158933. doi: 10.3389/fpls.2023.1158933 37025141 PMC10070872

[B46] Shoaib M. Hussain T. Shah B. Park S. H. (2022). Deep learning-based segmentation and classi fi cation of leaf images for detection of tomato plant disease 1–18. doi: 10.3389/fpls.2022.1031748 PMC9585275 36275583

[B47] Shovon M. S. H. Mozumder S. J. Pal O. K. Mridha M. F. Asai N. Shin J. (2023). PlantDet: A robust multi-model ensemble method based on deep learning for plant disease detection. IEEE Access. 1, 34–47. doi: 10.1109/ACCESS.2023.3264835

[B48] Sourav M. S. U. Wang H. (2023). Intelligent identification of jute pests based on transfer learning and deep convolutional neural networks. Neural Process. Lett. 55, 2193–2210. doi: 10.1007/s11063-022-10978-4 PMC9376051 35990859

[B49] Sun C. He Z. Lin H. Cai L. Cai H. Gao M. (2023). Anomaly detection of power battery pack using gated recurrent units based variational autoencoder. Appl. Soft Comput. 132, 109903. doi: 10.1016/j.asoc.2022.109903

[B50] Too E. C. Yujian L. Njuki S. Yingchun L. (2019). A comparative study of fine-tuning deep learning models for plant disease identification. Comput. Electron. Agric. 161, 272–279. doi: 10.1016/j.compag.2018.03.032

[B51] Turkoglu M. Yanikoğlu B. Hanbay D. (2022). PlantDiseaseNet: Convolutional neural network ensemble for plant disease and pest detection. Signal Image Video Process. 16 (2), 301–309. doi: 10.1007/s11760-021-01909-2

[B52] Vishnoi V. K. Kumar K. Kumar B. Mohan S. Khan A. A. (2023). Detection of apple plant diseases using leaf images through convolutional neural network. IEEE Access 11, 6594–6609. doi: 10.1109/ACCESS.2022.3232917

[B53] Wang X. (2022). Vehicle image detection method using deep learning in UAV video. Comput. Intell. Neurosci. 2022. doi: 10.1155/2022/8202535 PMC8847017 35178081

[B54] Wang Q. Qi F. Sun M. Qu J. Xue J. (2019). Identification of tomato disease types and detection of infected areas based on deep convolutional neural networks and object detection techniques. Comput. Intell. Neurosci. 2019, 1–5. doi: 10.1155/2019/9142753 PMC6942764 31933623

[B55] Xiong H. Li J. Wang T. Zhang F. Wang Z. (2024). EResNet-SVM: an overfitting-relieved deep learning model for recognition of plant diseases and pests. J. Sci. Food Agric. 2, 60–73. doi: 10.1002/jsfa.v104.10 38483173

[B56] Zainab A. Syed D. Al-Thani D. (2019). “Deployment of deep learning models to mobile devices for spam classification,” in Proc. - 2019 IEEE 1st Int. Conf. Cogn. Mach. Intell. CogMI 2019. IEEE, 112–117.

[B57] Zhang X. Bu J. Zhou X. Wang X. (2023). Automatic pest identification system in the greenhouse based on deep learning and machine vision. Front. Plant Sci. 14, 1255719. doi: 10.3389/fpls.2023.1255719 37841606 PMC10568774

[B58] Zhang S. Huang W. Zhang C. (2019). Three-channel convolutional neural networks for vegetable leaf disease recognition. Cogn. Syst. Res. 53, 31–41. doi: 10.1016/j.cogsys.2018.04.006

[B59] Zhang S. Zhang C. (2023). Modified u-net for plant diseased leaf image segmentation. Comput. Electron. Agric. 204, 107511. doi: 10.1016/j.compag.2022.107511

[B60] Zilvan V. Ramdan A. Suryawati E. Kusumo R. B. S. Krisnandi D. Pardede H. F. (2019). “Denoising convolutional variational autoencoders-based feature learning for automatic detection of plant diseases,” in 2019 3rd International Conference on Informatics and Computational Sciences (ICICoS). 1–6 (IEEE). doi: 10.1109/ICICoS48119.2019.8982494

